# Enhancement of gefitinib-induced growth inhibition by *Marsdenia tenacissima* extract in non-small cell lung cancer cells expressing wild or mutant EGFR

**DOI:** 10.1186/1472-6882-14-165

**Published:** 2014-05-22

**Authors:** Shu-Yan Han, Hui-Rong Ding, Wei Zhao, Fei Teng, Ping-Ping Li

**Affiliations:** 1 Key Laboratory of Carcinogenesis and Translational Research (Ministry of Education), Department of Integration of Chinese and Western Medicine, Peking University Cancer Hospital & Institute, No. 52 Fucheng Road, 100142 Haidian District, Beijing, P.R. China; 2 Central Laboratory of Biochemistry and Molecular Biology, Haidian District, P.R. China; 3 Department of Cell Biology, Peking University Cancer Hospital & Institute, 100142 Haidian District, Beijing, P.R. China

**Keywords:** *Marsdenia tenacissima* extract (MTE), Gefitinib, Non-small cell lung cancer (NSCLC), Combination, EGFR related pathway

## Abstract

**Background:**

Non-small cell lung cancer (NSCLC) expressed high levels of epidermal growth factor receptor (EGFR). Gefitinib (Iressa) has demonstrated clinical efficacy in NSCLC patients harboring EGFR mutations or refractory to chemotherapy. However, most of NSCLC patients are with wild type EGFR, and showed limited response to gefitinib. Therefore, to develop new effective therapeutic interventions for NSCLC is still required. Our previous study showed *Marsdenia tenacissima* extract (MTE) restored gefitinib efficacy in the resistant NSCLC cells, but whether MTE acts in the gefitinib-sensitive NSCLC cells is the same as it in the resistant one is unknown.

**Methods:**

Dose response curves for gefitinib and MTE were generated for two sensitive NSCLC cell lines with mutant or wild type EGFR status. Three different sequential combinations of MTE and gefitinib on cell growth were evaluated using IC_50_ and Combination Index approaches. The flow cytometric method was used to detect cell apoptosis and cell cycle profile. The impact of MTE combined with gefitinib on cell molecular network response was studied by Western blotting.

**Results:**

Unlike in the resistant NSCLC cells, our results revealed that low cytotoxic dose of MTE (8 mg/ml) combined gefitinib with three different schedules synergistically or additively enhanced the growth inhibition of gefitinib. Among which, MTE → MTE + gefitinib treatment was the most effective one. MTE markedly prompted cell cycle arrest and apoptosis caused by gefitinib both in EGFR mutant (HCC827) and wild type of NSCLC cells (H292). The Western blotting results showed that MTE → MTE + gefitinib treatment further enhanced the suppression of gefitinib on cell growth and apoptosis pathway such as ERK1/2 and PI3K/Akt/mTOR. This combination also blocked the activation of EGFR and c-Met which have cross-talk with each other. Unlike in gefitinib-resistant NSCLC cells, MTE alone also demonstrated certain unexpected modulation on EGFR related cell signal pathways in the sensitive cells.

**Conclusion:**

Our results suggest that MTE is a promising herbal medicine to improve gefitinib efficacy in NSCLC regardless of EGFR status. However, why MTE acted differently between gefitinib-sensitive and -resistant NSCLC cells needs a further research.

## Background

Lung cancer is the leading cause of cancer death worldwide. The high mortality of lung cancer is related to the fact that most patients present with metastatic disease for which there is no curative therapy. Non-small cell lung cancer (NSCLC) accounts for 75% - 80% of all lung cancers [1]. Epidermal growth factor receptor (EGFR) is a family member of EGF-related tyrosine kinase receptors, and expressed at high levels in many cancer cell types, including NSCLC [2]. This leads to inappropriate activation of the downstream signalling cascade, eventually leading to uncontrolled cell proliferation [3]. Studies showed EGFR is overexpressed in up to 80% of NSCLC and become a promising target for anti-cancer therapy [4,5].

An orally active tyrosine kinase inhibitor (TKI), gefitinib (ZD1839, Iressa), competes with ATP for the binding sites at tyrosine kinase domain, thereby dampening the phosphorylation and activation of EGFR so as to the downstream signaling network [6,7]. Gefitinib has been shown to significantly improve progression-free survival and used extensively for the first-line therapy in advanced NSCLC patients harboring EGFR mutations [8,9]. Exon 19 deletion mutations and L858 mutation in exon 21 of EGFR increase gefitinib sensitivity in NSCLC [10,11]. However, only 10% - 20% NSCLC patients with wild type of EGFR responded to gefitinib [12,13]. Moreover, clinical study revealed that NSCLC patients with EGFR mutations have a significantly longer survival than those with wild-type EGFR when treated with EGFR TKIs [14]. Despite experiencing dramatic clinical responses, patients who initially respond to gefitinib eventually develop progressive disease or incomplete cross-resistance to the currently available EGFR-TKIs like erlotinib [15,16]. Therefore, how to improve gefitinib efficacy and let more NSCLC patietns gain benefit from TKI therapy is still the goal of physicians and researchers.

*Marsdenia tenacissima* (Roxb.) Wight et Arn., which mainly produced in Yunnan province of China, has long been used as a remedy to treat cancer in China [17]. More than 40 C-21 steroidal glycosides have been isolated from the stem of *M. tenacissima*[18]. C-21 steroids have previously been shown to be cytotoxic in cancer cell lines [19] suggesting that C-21 steroids are responsible for the anti-cancer activities of *M. tenacissima*. Thirteen of the C-21 steroid compounds were identified in MTE by HPLC-MS/MS analysis [20]. Xiao-Ai-Ping injection, a water extract of *M. tenacissima*, has been proposed as a potential agent in the management of tumors, which is clinically effective in treatment of NSCLC when combined with chemotherapy [21-23]. Commonly, the treatment dosage of *Marsdenia tenacissima* extract (MTE) for cancer patients is between 20 ml to 100 ml (equals to 20 ~ 100 g crude drug) per day according to the manufacture’s instruction. According to our previous study, MTE restores gefitinib sensitivity in the resistant NSCLC cells and the mechanisms may be partially due to the down-regulation of PI3K/Akt/mTOR and ERK1/2 and inhibition of c-Met phosphorylation [20]. However, whether MTE could enhance gefitinib efficacy in the sensitive NSCLC cells is unknown, and whether the mode of action of MTE show difference or just the same in gefitinib-sensitive and -resistant NSCLC cells.

Therefore, the present study was to evaluate the regimen of MTE sequential combination with gefitinib against gefitinib-sensitive NSCLC cells, HCC827 (EGFR mutant) and H292 (wild type EGFR), and to seek the possible mechanisms may involve.

## Methods

### Cell culture

Human NSCLC cell lines HCC827 (epithelial adenocarcinoma) and H292 (alveolar epithelial carcinoma) were purchased from American Type Culture Collection (Manassas, Virginia, USA). H292 cells contains wild-type EGFR, whereas HCC827 bearing EGFR exon 19 deletion [24]. Cells were maintained in RPMI-1640 (Gibco) supplemented with 10% heat-inactivated fetal bovine serum (FBS) (Gibco), 100 units/ml penicillin, and 100 μg/ml streptomycin at 37°C and 5% CO_2_.

### Drug treatment

Gefitinib was purchased from AstraZeneca (Cheshire, UK). Stock solution was prepared in dimethyl sulfoxide (DMSO) at 20 mM and stored at −20°C. MTE (*M. tenacissima* extract, trade name: Xiao-Ai-Ping injection) (1 g crude/ml) was produced by SanHome Pharmaceutical Co., Ltd (NanJing, China). When the cells were treated with MTE or gefitinib, the MTE solvent or 0.5% DMSO was used as the negative control accordingly.

### Antibodies and reagents

The specific antibody of EGFR, p-EGFR (Tyr1068), p-PI3K, Akt, mTOR, p-mTOR (Ser2448), Met, p-Met (Y1234/1235), ERK1/2, p-ERK1/2, β-actin and β-tubulin were purchased from Cell Signaling Technology (Beverly, MA). PI3K and p-Akt (Ser473) were obtained from ABcam (Cambridge, UK). The enhanced chemiluminescence (ECL) system was from Millipore (Millipore, MA, USA). Epidermal growth factor (EGF) was purchased from Biosource International Inc. (Camarillo, CA) and dissolved in phosphate-buffered saline (PBS).

### Cell viability assay

The cell viability was measured by MTT assay. In brief, cells were plated in 96-well culture plates at the density of 1 ~ 1.5 × 10^4^ per well in complete medium. After 24 h incubation, cells were treated with gefitinib (0.001 ~ 50 μM) or MTE (0.5 ~ 500 mg/ml) for 72 h. The optical density at 570 nm was measured and the IC_50_ value was calculated based on the non-linear regression fit method by Graphpad Prism 4.0 software (San Diego, CA).

### Sequential combination effect evaluation

Cells were plated in 96-well culture plates and incubated for 24 h, then started treatment by following three different sequential combinations described previously [20,25]: (1) first pretreated cells with MTE for 12 h and then added gefitinib to MTE-containing medium for another 72 h (M → M + G); (2) MTE and gefitinib were added concurrently to the medium and incubated for 72 h (M + G); (3) first pretreated with gefitinib for 12 h and then added MTE to gefitinib-containing medium for another 72 h (G → G + M). Each sequential combination of MTE and gefitinib was characterized by a combination index (CI) as described by Chou [26] and calculated with CompuSyn (ComboSyn, Inc., Paramus, NJ, USA). The CI values were interpreted as follows: CI: 0.1-0.3 strong synergism, CI: 0.3-0.7 synergism, CI: 0.7-0.85 moderate synergism, CI: 0.85-0.9 slight synergism, CI: 0.9-1.1 nearly additive, and CI > 1.1 antagonism.

### Cell cycle analysis

H292 or HCC827 cells were pretreated with MTE for 12 h and then co-treated with or without gefitinib for another 72 h. Cells were detached with 0.25% EDTA-trypsin (Gibco), washed with PBS and fixed with ice-cold 70% ethanol overnight at 4°C. Cells were washed with PBS and incubated with RNase A (100 μg/ml) at 37°C for 30 min. DNA was labeled with PI (50 μg/ml) and the fluorescence was measured with a FACScalibur flow cytometer (Becton Dickinson). Data collection and analysis of the cell cycle distribution were performed using CellQuest and the Modfit software (Becton Dickinson).

### Detection of apoptosis by flow cytometry

H292 or HCC827 cells were pretreated with MTE for 12 h and then co-treated with or without gefitinib for another 72 h. Floating and adherent cells were collected and suspended in PBS, labeled with Annexin V and propidiumiodide (PI) following the manufacturer’s instruction (Biosea Biotechnology, Beijing, China). Flow cytometry (Bection Dikinson, USA) was used to assess the apoptotic cells. The quantitation of apoptotic cells was calculated by CellQuest software.

### Western blot analysis

After different drug treatment, the cells were stimulated with 10 ng/ml EGF for 15 min before harvesting, washed twice with cold PBS and lysed with RIPA buffer that containing protease and phosphotase inhibitors cocktail (Roche, UK). The supernatants were collected after centrifugation at 12000 rpm for 20 min. The protein was applied to polyacrylamide gel electrophoresis (SDS-PAGE), transferred to a PVDF membrane, and then detected by the proper primary and secondary antibodies before visualization with a chemiluminescence kit. The intensity of blot signals was quantitated using ImageQuant TL analysis software (General Electric, UK).

### Statistical analysis

All data were expressed as means ± standard error of the mean (SEM) obtained from at least three independent experiments. Statistical comparisons between experimental and control groups were assessed by using the Student’s *t*-test. *P* < 0.05 was considered statistically significant.

## Results

### Anti-proliferative activity of gefitinib and MTE in NSCLC cells

**Figure 1:**
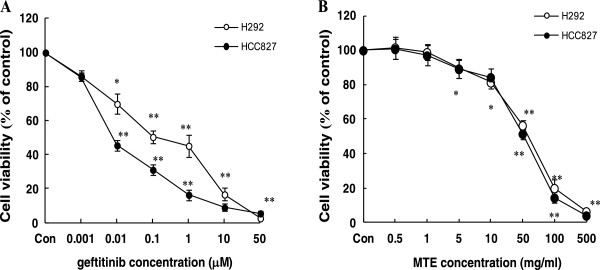
**Treatment of non-small cell lung cancer cells with gefitinib or MTE (*M. tenacissima *extract) reduced their proliferation potential. (A)** H292 and HCC827 cells were treated with the indicated concentrations of gefitinib for 72 h. **(B)** H292 and HCC827 cells were treated with the indicated concentrations of MTE for 72 h. Cells viability was determined by using the MTT assay as described in the Methods section. The data are expressed in terms of percent of control cells as the means ± SEM. The experiments were repeated at least three times. ^*^P < 0.05, ^**^P < 0.01 vs. control group.

We examined the in vitro cell growth inhibition of gefitinib or MTE on NSCLC cells by using MTT assay. After treatment for 72 h, the IC_50_ value for gefitinib was 0.166 μM for H292 cells (EGFR wild type), and 0.015 μM for HCC827 cells (EGFR exon 19 deletion) (Figure 1A), indicating they are gefitinib-sensitive cell lines. H292 carrying wild-type EGFR but highly sensitive to gefitinib, suggesting that there exists off-target(s) of gefitinib in addition to the commonly accepted target EGFR, which deserves a further investigation. When exposed to MTE, these two cell lines showed a similar dose-dependent inhibition manner on cell viability regardless of the EGFR status (Figure 1B). The IC_50_ value of MTE was 46.62 mg/ml for H292 cells and 48.78 mg/ml for HCC827 cells, respectively.

### Sequential-combined inhibitory effect of gefitinib and MTE in NSCLC cells

**Figure 2:**
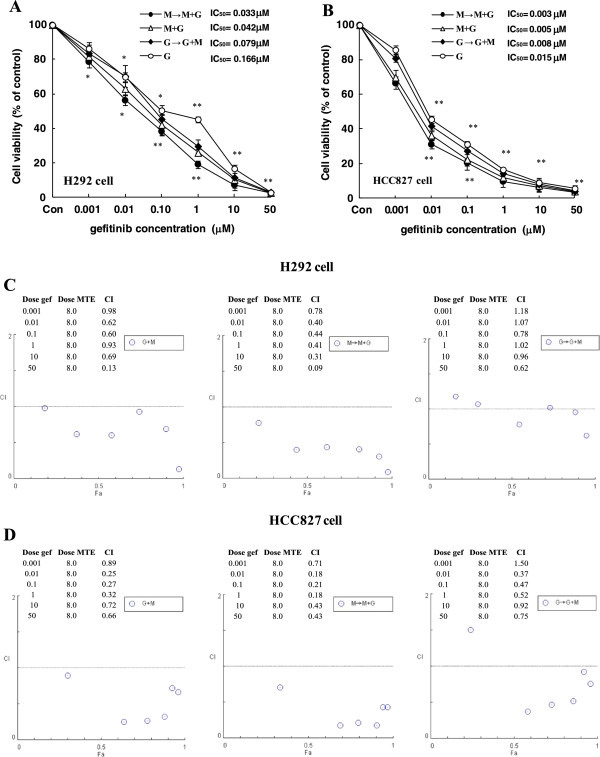
**MTE (*M. tenacissima *extract) enhances gefitinib induced cytotoxicity in gefitinib-sensitive non-small cell lung cancer cell lines.** H292 **(A)** or HCC827 **(B)** cells were incubated with increasing concentrations of gefitinib and MTE alone or different schedule combinations. Treatment schedule: (1) M → M + G, MTE pretreated for 12 h, then M + G (MTE + gefitinib) for another 72 h. (2) M + G, MTE and gefitinib concomitantly treated for 72 h. **(3)** G → G + M, gefitinib pretreated for 12 h, then G + M (gefitinib + MTE) for another 72 h. Growth assays were performed by MTT assay and IC50 values were calculated by Graphpad Prism 5.0 software (San Diego, CA, USA). CI values for the combinations of gefitinib and MTE in H292 **(C)** and HCC827 **(D)** cells were calculated using the Calcusyn software (Cambridge, UK), as described in the Methods section. Each data point represented the mean of 3 to 4 replicates; *r* values for all curves were >0.95. ^*^P < 0.05, ^**^P < 0.01 vs. control group.

The effect of sequential combination of gefitinib and MTE was examined in H292 and HCC827 cells. In the following experiments, MTE is set at a concentration of 8 mg/ml for HCC827 and H292 cells (approximately IC_15_ value). At this concentration, MTE showed only weak inhibition of cell growth with the cell viability approximately 90%. Compared with gefitinib or MTE alone, all cells treated with gefitinib plus MTE exhibited decreased viability (Figure 2A and B). The combination index (CI) values were calculated by CompuSyn software as described in Materials and Methods. Linear correlation coefficients (r values) for all curves were >0.95, indicating a high goodness of fit. As shown in Figure 2C, the CI values in H292 cells treated by G + M and M → M + G were all <1, indicating that there was a synergistic interaction between gefitinib and MTE in these two sequential combinations. The CI values in G → G + M treated H292 cells predicted antagonism at the lowest gefitinib concentration but nearly additive to synergistic effects at other high concentrations. In Figure 2D, CI values in HCC827 cells treated by three different sequential combinations had same tendency as they were in H292 cells, but the synergism effects were even more pronounced. Among the three different combinations, the M → M + G treatment was potent over the other two sequential combinations both in H292 and HCC827 cells, indicating this was the preferred combination of gefitinib and MTE.

### MTE affected gefitinib on cell cycle distribution

**Figure 3:**
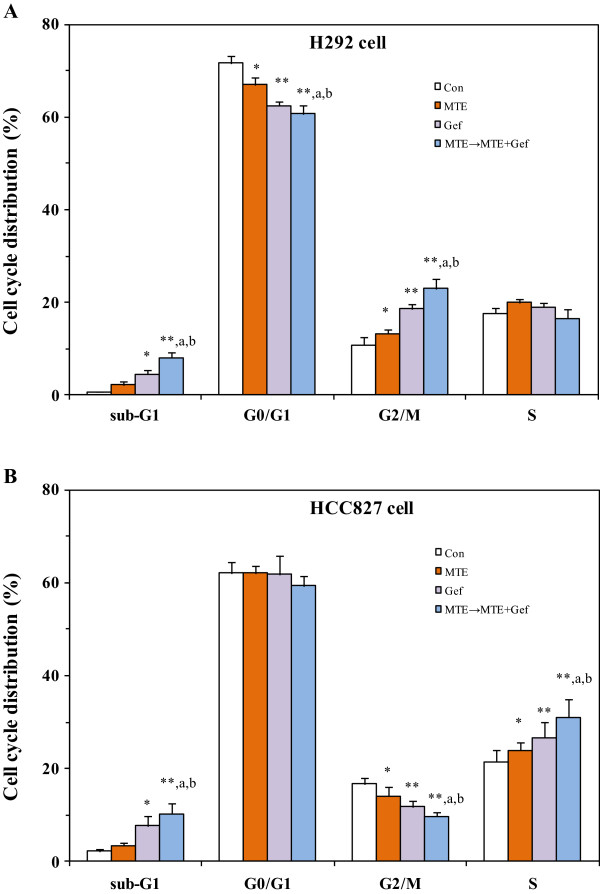
**MTE (*M. tenacissima *extract) enhances gefitinib induced delay in cell cycle in non-small cell lung cancer cell lines H292 (A) and HCC827 (B).** Cells were treated with 1 μM gefitinib, 8 mg/ml MTE, and their combination (MTE → MTE + Gef, M → M + G) in 1% FBS culture medium for 72 h, then harvested, ethanol fixed and labeled with PI for the analysis of cell cycle by FACS analysis. Each data presented as the means ± SEM of three experiments. ^*^P < 0.05; ^**^P < 0.01*vs* vehicle control group, ^a^*P* < 0.05 *vs* MTE treated group, ^b^*P* < 0.05 *vs* gefitinib treated group.

The effect of MTE combined with gefitinib on cell cycle distribution was examined with flow cytometry. As shown in Figure 3A, MTE treatment caused increase at G2/M phase (from 10.7% to 13.2%) and decrease at G0/G1 phase (from 71.7% to 67.0%) in H292 cells when compared with control group. The fraction cells in G2/M phase were significantly elevated to 18.6% and the proportion of G0/G1 phase cells was depressed to 62.5% by gefitinib alone. Moreover, MTE → MTE + Gef treatment further raised the percentage of G2/M phase cells to 22.9% and reduced G0/G1 phase cells to 60.7%. The results above suggested that MTE enhanced gefitinib induced G2/M arrest in H292 cells.

Compared with control group, it showed that HCC827 cells treated with MTE induced accumulation in the S phase of cell cycle from 21.5% to 23.9% while G2/M phase cells decreased from 16.7% to 14.1%. 1 μM gefitinib treated HCC827 cells were markedly accumulated in the S phase of cell cycle to 26.6% and the G2/M phase cells were decreased to 11.7%. The percentage of G2/M-phase cells was further decreased to 9.6% in the MTE → MTE + Gef group, and induced a significant cell population in S phase to 31.0% (Figure 3B). This finding indicates that cell cycle distribution was blocked significantly in the S phase when HCC827 cells were treated with MTE combined with gefitinib. Accumulation in the sub-G1 phase of cell cycle were observed both in H292 and HCC827 cells, suggesting the sequential events of cell cycle arrest followed by apoptosis.

### MTE enhanced gefitinib-induced apoptosis in NSCLC cells

**Figure 4:**
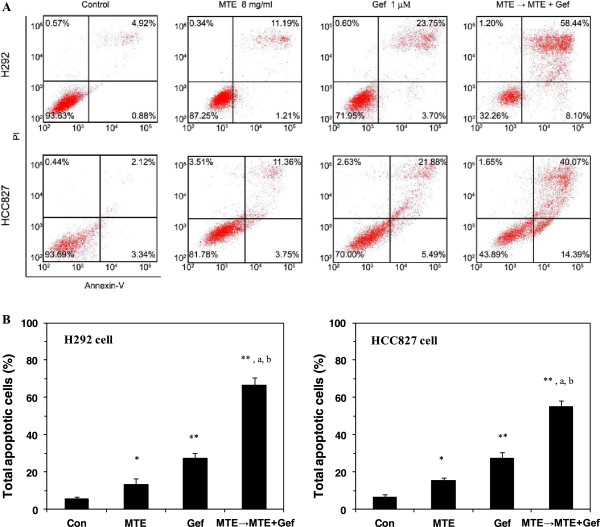
**MTE (*M. tenacissima *extract) prompts apoptosis induced by gefitinib in non-small cell lung cancer cells. (A)** H292 and HCC827 cells were treated with 1 μM gefitinib, 8 mg/ml MTE, and their combination (MTE → MTE + Gef, M → M + G) in 1% FBS culture medium for 72 h, then harvested and labeled with Annexin V-PI for the analysis of apoptotic cells by FACS analysis. The lower right quadrant and the upper right quadrant of the FACS histograms indicate the percentage of early and late apoptotic cells, respectively. **(B)** Total percentages of apoptotic cells in each treatment group are summarized with data presented as the means ± SEM of three experiments. ^*^P < 0.05; ^**^P < 0.01*vs* vehicle control group, ^a^*P* < 0.05 *vs* MTE treated group, ^b^*P* < 0.05 *vs* gefitinib treated group.

To examine the mechanism of inhibitory effect of MTE combined with gefitinib, cell apoptosis was performed by flow cytometry. The final concentration of gefitinib used in the treatments was 1 μM because it closes to the serum level observed in patients being treated with gefitinib [27]. Apoptotic cell death was determined in terms of early- or late-stage apoptotic cells, which are shown respectively in the lower right and upper right quadrants of the FACS histograms (Figure 4A). Treatment of H292 and HCC827 cells with gefitinib and MTE alone or in combination induced a substantial fraction of apoptosis. The percentage of total apoptotic cells (including early and late stage) in H292 cells after different treatment was as follows: 5.8% (vehicle-treated control), 12.4% (MTE 8 mg/ml), 27.4% (gefitinib 1 μM), and 66.5% (MTE → MTE + Gef, M → M + G) (Figure 4). Similar tendency of apoptosis was observed in HCC827 cells as shown in Figure 4A and 4B. The combined treatment of MTE and gefitinib further enhanced cell apoptosis that induced by gefitinib in H292 and HCC827 cell lines, and showed significant difference with each drug alone (*P* < 0.05). The results revealed that M → M + G treatment was effective in cell apoptosis induction.

### MTE affected EGFR downstream pathways inhibition by gefitinib

**Figure 5:**
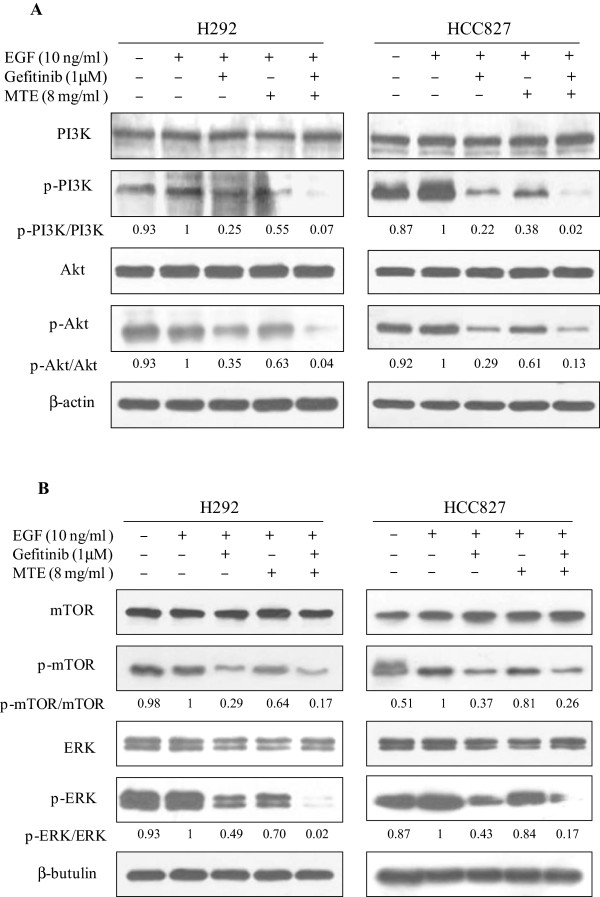
**MTE (*M. tenacissima *extract) enhances the inhibition of gefitinib on PI3K/Akt/mTOR and ERK1/2 signaling cascade in non-small cell lung cancer cells.** H292 and HCC827 cells were treated with 1 μM gefitinib, 8 mg/ml MTE, and their combination (MTE → MTE + Gef, M → M + G) in 1% FBS culture medium for 6 h. Cells were stimulates with 10 ng/ml EGF for 15 min before harvest. Cells were lysed and cellular extracts (20 μg protein) were separated on SDS-PAGE gel and transferred to PVDF membranes. **(A)** Membranes were probed with PI3K, phospho-PI3K, Akt and phospho-Akt; **(B)** Membranes were probed with mTOR, phospho-mTOR, ERK1/2 and phospho-ERK1/2. Blots are representatives of three independent experiments. Relative density of proteins were calculated and normalized based on β-tubulin or β-actin.

The major pathways activated by EGFR-TK are the Ras-Raf-MEK-ERK pathway (leading to cell growth), the mTOR pathway (leading to protein synthesis), and the PI3K/Akt pathway (leading to cell survival by blocking apoptosis). To investigate the combined effects of MTE and gefitinib on EGFR-dependent signaling pathways, cells were stimulated with 10 ng/ml EGF for 15 minutes before harvest. We found EGF stimulated the activation of PI3K/Akt/mTOR and ERK1/2, and their phosphorylation extent is much higher in HCC827 cell (EGFR mutant) than H292 cells (EGFR wide type) (Figure 5). The activation of EGFR downstream pathways was strongly inhibited by gefitinib treatment. Treated with MTE moderately reduced PI3K/Akt/mTOR and ERK1/2 phosphorylation with a similar profile in H292 and HCC827 cells. Moreover, the inhibition was further enhanced by treatment of M → M + G and blocked PI3K/Akt/mTOR and ERK1/2 signaling pathways.

### MTE enhanced EGFR and c-Met inhibition by gefitinib

**Figure 6:**
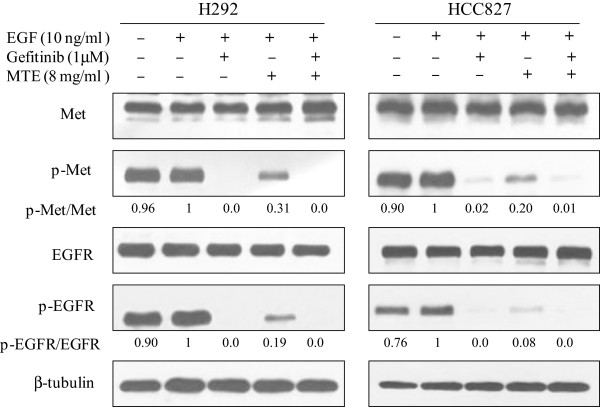
**MTE (*M. tenacissima *extract) combined with gefitinib reduces EGFR and Met crosstalk in non-small cell lung cancer cells.** H292 and HCC827 cells were treated with 1 μM gefitinib, 8 mg/ml MTE, and their combination (MTE → MTE + Gef) in 1% FBS culture medium for 6 h. Cells were stimulates with 10 ng/ml EGF for 15 min before harvest. Cells were lysed and cellular extracts (20 μg protein) were separated on SDS-PAGE gel and transferred to PVDF membranes. Membranes were sequentially probed with Met, phospho-Met, EGFR and phospho-EGFR. β-tubulin was served as a loading control. Blots are representatives of three independent experiments.

c-Met and EGFR receptors are widely expressed in cancer cells. The c-Met receptor tyrosine kinase has a central role in the survival of cancer cell and has been identified as a novel promising target for NSCLC [28]. It demonstrated that EGFR activation contributes to c-Met tyrosine phosphorylation [29]. Our results showed EGF slightly stimulated the phosphorylation of c-Met and EGFR in H292 and HCC827 cells (Figure 6). However, gefitinib alone or combined with MTE totally blocked the phosphorylation of c-Met and EGFR in these two NSCLC cells. This is in consistent with other research that gefitinib simultaneously inhibited both EGFR and c-Met phosphorylation in gefitinib-sensitive NSCLC cells [30]. Moreover, the activation of EGFR and c-Met was also significantly inhibited by MTE treatment alone as shown in Figure 6. The inhibition on EGFR and c-Met phosphorylation may partly explain the underlying mechanisms of MTE on EGFR-TKI sensitive NSCLC.

## Discussion

The two primary signaling pathways activated by EGFR include the Ras/Raf/MEK/ERK and the PI3K/Akt axes [31]. In the understanding of the nature of NSCLC carcinogenesis, EGFR-TKIs become an innovative approach for NSCLC patients to achieve more effective treatment. Gefitinib exerted anti-tumor effect by inhibiting EGFR-driven signaling activation such as Akt and ERK1/2 in the sensitive NSCLC cells [32]. In our research, gefitinib inhibited EGFR and its downstream signaling pathways such as PI3K/Akt/mTOR and ERK in gefitinib-sensitive NSCLC cells, HCC827 and H292, as expected. However, in the resistant NSCLC cells, EGFR is not a survival factor and other factor driven cell survival following activation of downstream signaling effectors was dominant [32].

Nowadays, the use of herbal medicines or compounds from them is becoming increasingly popular, and provides alternative treatment options for cancer patients [33]. It has been demonstrated that curcumin potentiates the antitumor activity of gefitinib in NSCLC cell lines and xenograft mice model [34]. Co-treatment with gefitinib and epigallocatechin gallate (EGCG), a green tea polyphenol, synergistically suppressed the metastatic ability of CAL-27 human oral squamous cell carcinoma cells [35]. We previously reported that *M. tenacissima* extract (MTE, Xiao-Ai-Ping injection) restored gefitinib sensitivity in the resistant NSCLC cells through synergistic inhibition of PI3K/Akt/mTOR, ERK1/2 activation and c-Met phosphorylation [20]. In the present study, human NSCLC cell line HCC827 and H292 cells were very sensitive to gefitinib with IC50 at 0.015 μM and 0.166 μM, respectively. MTE was also cytotoxic to HCC827 and H292 cells when treated alone. Compared to the antitumor effect of each reagent alone, the combined gefitinib and MTE treatment synergistically or additively suppressing cell viability regardless of the sequential difference. Among which, the MTE → MTE + Gef treatment showed more potent on decreasing cell growth than the other combinations, concurrent administration and Gef → Gef + MTE. Furthermore, the enhanced cell proliferation inhibition by MTE → MTE + gefitinib sequential combination was over each agent alone. This is in accordance with our previous result that MTE → MTE + Gef had potential effect against gefitinib-resistant NSCLC cell lines [20]. However, it was very interesting that the Gef → Gef + MTE schedule was also effective in reducing proliferation of H292 and HCC827 cells, whereas an antagonistic effect was observed in the resistant cells, H460 and H1975. The underlying reasons for Gef →Gef + MTE acts different in gefitinib-resistant and -sensitive NSCLC cells deserve a further research. MTE also could enhance the tumor growth inhibitory effect of erlotinib, another common used TKI, by the three different combination procedures (see Additional file 1). In other studies, the exhibited synergistic or additive effects by different reagents combinations were also demonstrated. Paclitaxel followed by gefitinib was regarded as an effective treatment combination for NSCLC cell lines harboring EGFR mutations [36], while schedule-dependent synergistic effect was seen in the combinations of gefitinib and irinotecan in lung cancer cell lines [25].

Flow cytometric data revealed MTE and gefitinib alone or in combination caused different cell cycle arrest in H292 and HCC827 cells. They clearly induced G2/M arrest in H292 cells but caused S phase block in HCC827 cells, and this discrepancy may due to cell characteristics difference. The combination of MTE → MTE + gefitinib enhanced cell cycle arrest and apoptosis that induced by gefitinib in NSCLC cell lines and showed significant difference with each drug alone (*P* < 0.05). Taken together, these findings in part suggest that MTE → MTE + Gef could inhibit the growth of H292 and HCC827 cells by inducing cell cycle arrest and apoptosis, and induction of apoptosis was seems to be more important in this study.

Over activation of ERK which is one of the main cell proliferation and survival factors. Studies showed that constitutive ERK1/2 activity in NSCLC cells promotes cellular survival and chemotherapeutic resistance [37]. In our research, the phosphorylation of ERK was significantly suppressed by gefitinib. MTE also decreased ERK activation to some extent. The combination of MTE → MTE + Gef treatment enhanced the inhibition of gefitinib on H292 and HCC827 cell growth. This prominent suppression of cell proliferation was correlated with an enhanced inhibition on ERK1/2 activation by the combination of MTE → MTE + Gef.

The PI3K/Akt/mTOR is an important intracellular signaling pathway in cell apoptosis, and this deregulated cascade is reported to contribute to lung cancer development and maintenance [38]. Frequent Akt activation and mTOR phosphorylation were found in 51% of NSCLC patients and in 74% of NSCLC cell lines [39]. NSCLC cell lines responsive to EGFR TKIs with growth arrest or apoptosis showed a down-regulation of the PI3K/Akt/mTOR pathway [40]. In this study, gefitinib prominently inhibited the activation of PI3K/Akt/mTOR. MTE alone also moderately decreased the phosphorylation of these signaling molecules at a relative low concentration used in the study (~IC_15_, 8 mg/ml). Furthermore, MTE → MTE + Gef treatment acted synergistically in disruption of the PI3K/Akt/mTOR signaling cascade and induction of apoptosis in H292 and HCC827 cells.

c-Met and EGFR receptors are widely expressed in cancer cells, and they have redundant effects on cell cycle progression, apoptosis, motility and metastasis and are potential targets for combination therapy. There is a cross-talk between the c-Met and EGFR signaling pathways in lung cancer [29]. EGFR and c-Met kinase activity is required for EGF-induced c-Met phosphorylation. The present study showed treatment with gefitinib abolished tyrosine phosphorylation of c-Met and EGFR as it demonstrated in other research [29,41]. Of course, the combination of MTE and gefitinib further decreased c-Met and EGFR phosphorylation. To our surprise, MTE alone at a relative minimal cytotoxic concentration also decreased c-Met and EGFR phosphorylation very strongly in the present study, suggesting it could inhibit both of these two growth factor receptors. However, MTE alone only showed inhibition tendency in the resistant cells according to our previous experiment [20]. The same phenomenon was also observed in above mentioned PI3K/Akt/mTOR pathway and ERK1/2. It suggests that MTE acted differently in gefitinib-sensitive and -resistant NSCLC cells. But what factors resulted in the difference of MTE activity is an interesting issue and needs a further study.

## Conclusion

In summary, we have found MTE and gefitinib acted synergistically or additively with different combinations to suppress gefitinib sensitive NSCLC cell growth over treatments with either agent alone. Meanwhile, the cell cycle arrest and apoptotic cells were greatly promoted by MTE → MTE + Gef treatment, the most powerful combination. The underlying mechanisms of this remarkable cell growth inhibition were mostly ascribed to the significant suppression of ERK1/2 and PI3K/Akt/mTOR. The blocked crosstalk of EGFR and c-Met was also contributed to the prominent anti-tumor effect of MTE → MTE + Gef treatment. However, there was no obvious difference between the effect of this combination in EGFR mutant cell line HCC827 and wild type cell line H292. Thus, our results suggest that MTE is a promising herbal medicine to improve gefitinib efficacy in NSCLC regardless of EGFR status. The different effect of MTE in gefitinib-sensitive and -resistant NSCLC cells needs further research.

## Abbreviations

MTE: *Marsdenia tenacissima* extract; EGFR: Epidermal growth factor receptor; NSCLC: Non-small cell lung cancer; CI: Combination index; ECL: Chemiluminescence; EGF: Epidermal growth factor; TKI: Tyrosine kinase inhibitor.

## Competing interests

The authors declare that they have no competing interest in the study.

## Authors’ contributions

SYH performed experiments, participated in experimental design and drafted the manuscript. HRD, WZ and FT carried out the experimental procedures and analyzed the data. PPL conceived of the study, participated in its design and coordination, and helped to draft the manuscript. All authors read and approved the final manuscript.

## Pre-publication history

The pre-publication history for this paper can be accessed here:

http://www.biomedcentral.com/1472-6882/14/165/prepub

## Supplementary Material

Additional file 1 **MTE (*M. tenacissima *** **extract) enhances erlotinib induced cytotoxicity in gefitinib-sensitive non-small cell lung cancer cell lines H292 (A) and HCC827 (B).** Treatment schedule: (1) M → M + E, MTE pretreated for 12 h, then M + E (MTE + erlotinib) for another 72 h. (2) M + E, MTE and erlotinib concomitantly treated for 72 h. (3) E → E + M, erlotinib pretreated for 12 h, then E + M (erlotinib + MTE) for another 72 h. CI values were calculated using the Calcusyn software (Cambridge, UK), as described in the Methods section. ^*^P < 0.05, ^**^P < 0.01 vs. control group. Click here for file
