# Supplementary material for: Enhancement of gefitinib-induced growth inhibition by Marsdenia tenacissima extract in non-small cell lung cancer cells expressing wild or mutant EGFR
Source: BMC Complement Altern Med. 2014 May 22;14:165. doi: 10.1186/1472-6882-14-165 (PMC4040364; doi:10.1186/1472-6882-14-165)
Supplement: Additional file 1 — MTE ( M. tenacissima extract) enhances erlotinib induced cytotoxicity in gefitinib-sensitive non-small cell lung cancer cell lines H292 (A) and HCC827 (B). Treatment schedule: (1) M → M + E, MTE pretreated for 12 h, then M + E (MTE + erlotinib) for another 72 h. (2) M + E, MTE and erlotinib concomitantly treated for 72 h. (3) E → E + M, erlotinib pretreated for 12 h, then E + M (erlotinib + MTE) for another 72 h. CI values were calculated using the Calcusyn software (Cambridge, UK), as described in the Methods section. *P < 0.05, **P < 0.01 vs. control group. [file 1472-6882-14-165-S1.docx]

**Additional file 1:** MTE (*M. tenacissima* extract) enhances erlotinib induced cytotoxicity in non-small cell lung cancer cell lines H292 (A) and HCC827 (B).
